# The impact of financial crisis and austerity policies in Andalusia, Spain: disentangling the mechanisms of social inequalities in health through the perceptions and experiences of experts and the general population

**DOI:** 10.1186/s12939-019-1013-3

**Published:** 2019-07-16

**Authors:** Javier Alvarez-Galvez, Victor Suarez-Lledo, Gloria Martinez-Cousinou, Eider Muniategui-Azkona, Auxiliadora Gonzalez-Portillo

**Affiliations:** 1 Department of Biomedicine, Biotechnology and Public Health, University of Cadiz, Cadiz, Spain; 2 Department of International Studies, Loyola University Andalusia, Seville, Spain; 3 Department of Social Work and Social Services, Pablo de Olavide University, Seville, Spain

**Keywords:** Financial crisis, Social determinants of health, Socioeconomic status, Health inequalities, Qualitative methods, Spain

## Abstract

**Background:**

Andalusia has been one of the regions most damaged by the economic crisis in Spain. A qualitative study of the effects of the economic crisis and austerity policies in this region has been conducted within the framework of the IMPACT-A project. This research seeks to analyse the perceived impact of the crisis upon the health of the Andalusian population through the first-hand discourses of professionals from the health and social sectors on the one hand, and citizens of different socioeconomic status (SES) on the other.

**Methods:**

A total of five focus groups and ten semi-structured interviews were conducted and analysed following an inductive process based on Grounded Theory (GT).

**Results:**

Our results show a general perception among professionals: the financial crisis has either directly or indirectly affected population health in Andalusia, though mostly impacting low-income individuals who were already at risk of social exclusion. Professionals’ perceptions have been confirmed through the discourses of citizens of a lower SES, which differ from those of middle and upper SES.

**Conclusion:**

Findings reveal some of the most salient consequences on the socially vulnerable groups and people at risk of social exclusion. In particular, our study highlights the importance of addressing three areas of priority action: mental health, unmet (basic and medical) needs, and decline in the health system.

## Background

Although all European countries have been affected by the recent financial crisis [1–3], the impact has been greater for Mediterranean countries such as Greece, Italy or Spain [4]. In Spain, this greater impact stems from the coincidence of the global financial crisis with the end of the so called “real estate bubble”, which had been one of the main economic forces in the country [4]. The same is true of the Troika-promoted austerity policies that have impacted Spanish social and health policies, as well as social services that are meant to protect families [5].

Together with the Canary Islands and Extremadura, Andalusia has been the Spanish region that most severely suffered the consequences of the crisis in terms of employment. During these years, Andalusia has been at the forefront of the Spanish regions in terms of the percentage of the population living in severe material poverty according data from the National Institute of Statistics [6]. Analysis of the health budget drawn up by the Andalusian Government for the years 2009 and 2015 shows that there was a drop of 1,400 million euros, representing a fall of 13.9% compared to the average 9% for Spain as a whole.

This situation profoundly changed the healthcare system. The austerity measures launched in 2009 included the closure of several health services and a reduction in the number of hospital beds and healthcare staff [4, 5, 7, 8]. Moreover, a co-payment fee for drug prescriptions for pensioners and high-income populations was introduced [9]. Finally, Statutory Order 16/2012 modified substantial elements of the Spanish healthcare system. From that moment on, health coverage shifted from being based on residency to depending on social security contributions.

In line with this, the Andalusian regional government also reduced healthcare spending during the crisis period. This action has resulted in the closing of healthcare infrastructure, a reduction in the number of hospital beds and the loss of about 7,265 jobs in the Andalusian Health Service [10]. Consequently, there has been an increase in waiting lists together with a progressive decline in the quality of healthcare services [8]. Primary healthcare services, which play a fundamental role when it comes to the prevention and early detection of many illnesses, have been especially affected [4].

Over the last few years, the number of studies conducted into the impact of the economic downturn and austerity policies on population health and the healthcare system has increased considerably both in Spain and Europe [5–7, 9, 11–17]. A relevant body of literature on this topic has adopted a descriptive perspective based on statistical data on population health [6, 15, 16, 18] or the evolution of health services [7, 13, 17]. However, less attention has been paid to understanding the specific problems and shared concerns of social groups that have experienced the context of economic recession and austerity [5], as well as possible solutions from the perspective of the different groups.

In an attempt to shed light on this gap in the literature, this research aims to explore the social perception of the impact of the economic crisis on health and the public health system in Andalusia. This study compiles the results of a qualitative study conducted as part of the IMPACT-A project (Socioeconomic Determinants of Health in Andalusia: Studying the Impact of the Economic Crisis on Andalusian Health, PRY140/20) [19]. The qualitative study includes two relevant aspects that have remained largely ignored thus far. On the one hand, the perceived impact of the economic crisis and austerity policies on professionals working in the healthcare system and the social sector; and, on the other hand, the perceived impact of these phenomena among the general population in Andalusia.

## Method

### Materials and method

This research was conducted during the months of November 2015 and June 2016 in three out of the eight provinces of Andalusia that were considered to be representative of the Andalusian geography: (1) Sevilla, which is the political and administrative capital of the region as well as the most populated province in Andalusia with a population of 1,939,775 inhabitants; (2) Cádiz, a middle-sized coastal province with a population of 1,239,889 inhabitants; and (3) Córdoba, which is an undersized inland province compared to the average Andalusian provinces, with 791,610 inhabitants.

In this study, interviews and focus groups were used to generate information. We chose these techniques inasmuch as the focus of the research (perceptions and discourses) led us to design a sufficiently flexible tool so as to explore the perceptions of the professionals we contacted, although limiting the information to a set of analytical dimensions linked to our research purposes (i.e. expert knowledge, socioeconomic problems, and unmet needs). All the interviews and focus groups were conducted individually by university professors with experience in qualitative research (JAG, AGP, GMC, EMA).

A total of 5 focus groups and 10 semi-structured interviews were conducted by 4 qualitative research experts throughout the region (Table 1). The average duration of the focus groups was 120 min and 35 min for interviews. Groups were internally homogenous, that is, for each of the focus groups only specific population groups were included according to the research goals. In FG1, men and women of lower socioeconomic status (SES) living in Seville were included; in FG2, middle SES (both men and women) residents in Jerez de la Frontera (Cádiz) were included; and in FG3, men and women of upper SES people living in Córdoba were included. On the other hand, focus groups 4 and 5 were composed of social intervention and health professionals, respectively. For both cases, we selected individuals of either sex who had worked for the last 10 years in public Andalusian institutions related to healthcare or the social sector, so they would have professional experience before the 2008 financial crisis. Socioeconomic focus groups (FG1–3) were conducted in local community centres of the chosen cities, and the FGs of professionals were held in their workplace (i.e. a primary care centre in Seville, and an NGO).

**Table 1:** The participants’ characteristics and identifiers

ID	Groups and interviews description	Total	Male	Female
FG1	Lower Social Class Population (men and women)	6	5	1
FG2	Middle Social Class Population (men and women)	8	3	5
FG3	Upper-Middle Social Class Population (men and women)	7	4	3
FG4	Professionals in the Social Intervention Field (men and women)	9	3	6
FG5	Experts on Health (men and women)	7	3	4
I1	General Practitioner (GP)	1	1	
I2	NGO Manager	1	1	
I3	Female caregiver of person affected by the crisis	1		1
I4	Expert Professor of Public Health	1	1	
I5	60-year-old woman, Middle Class, health issues	1		1
I6	40-year-old man, Lower Class, financial issues	1	1	
I7	55-year-old man, Upper Class, no health issues	1	1	
I8	25-year-old woman, Middle Class, no health issues	1		1
I9	34-year-old man, Lower Class, financial issues	1	1	
I10	50-year-old woman, Middle Class, no health issues	1		1
Total		47	24	23

FGs and interviews were conducted in Spanish and subsequently translated into English. The translation process was also reviewed by a professional copy-editing service.

### Sample selection

In order to capture the perceptions of both healthcare professionals and populations from different socioeconomic strata, the participants were selected by means of a snow ball (or chain) sampling process, wherein new study subjects recruited additional subjects from among their acquaintances. In the case of professionals, the researchers directly contacted public healthcare and social sector institutions via phone or email to request contact with professionals who had more than 10 years of professional experience. In the case of the general population, the researchers visited different healthcare and community centres located in Andalusian districts of varying socioeconomic levels to contact health and social services users who were waiting to be attended, or indirectly find some relatives or friends that met the inclusion criteria to saturate the information available. The different socioeconomic levels were evaluated based on the districts of origin. Once this list of potential participants was drawn up, focus groups were formed considering three key components: the participants in any given group did not know each other so as to avoid elements of distortion; both men and women were represented in each group so that they could share their experiences of the context of crisis and economic austerity from different perspectives; and the participants had to be available to meet on a specific date.

To complete the information obtained from the focus groups, 4 semi-structured interviews were held with health professionals: a general practitioner (GP), an NGO manager, a caregiver, and an expert professor of public health. In addition, 6 interviews were conducted with users or potential users of the public healthcare system to obtain first hand discourse on the impact of the crisis on their health: 2 low-income individuals facing financial issues; 1 middle-income individual with health problems, and 2 from the same socioeconomic level without health problems; and 1 upper SES individual with no health problems.

The people who were interviewed did not participate in the focus groups. Their contact was facilitated either by institutions associated with the health and social sectors that were consulted in order to form the focus groups, or by the actual participants of the focus groups (Table 1).

Certain difficulties were encountered in the sampling process to select individuals from the lower social classes, who often initially refused to participate in the study, and in particular healthcare professionals, who were generally unavailable due to their working schedules. However, once every informant agreed to participate in the study, they all demonstrated a clear disposition and interest to contribute to a research topic that they considered fundamental for their working and living conditions.

### Interview guide

When we designed the questions for the interviews and focus groups, we decided to follow the Kvale framework [20], which distinguishes between two types of questions. On the one hand, thematic research questions (TRQ), which encompassed the core thematic points about which we wanted to inquire and to which we gave a temporal dimension (past, present, future). On the other hand, dynamic interview questions (DIQ), which are consistent with the translation of those general topics into more colloquial language so that it can be used and understood by the interviewee during the development of the interview. Various dynamic questions can correspond to one thematic research question, as shown in Table 2.

**Table 2:** Research questions covered in focus groups and interviews

Thematic Research Questions (TRQ)	Dynamic Interview Questions (DIQ)
TRQ1. SES before and after the crisis.	DIQ1. What was your life like before the crisis? Have you experienced any change? What were the reasons?
	DIQ2. Do you believe the crisis has affected the life of Andalusians? What specific aspects of daily life do you perceive to be affected the most by the crisis?
	DIQ3. And for you and the people in your immediate environment, how has the crisis affected all of you?
TRQ2. Health impact of the crisis	DIQ4. Do you believe the crisis has had health consequences for Andalusians? In what ways?
	DIQ5. Can you imagine any possible ways to avoid these situations? Any solutions?
	DIQ6. And with regards to mental health, do you think that Andalusian people have been affected in any way?
	DIQ7. How has the crisis affected the Andalusian public healthcare system?
TRQ3. Future health expectations	DIQ8. What do you think the health of Andalusians will be like in the future?
	DIQ9. Alternative and/or proposal for improved health measures.

Both the interviews and the focus groups were recorded, transcribed, and approved in agreement with the informed consent of each participant. The participants’ personal information together with any identifying elements have been systematically anonymised in the final materials.

### Information and data analysis

**Fig. 1:**
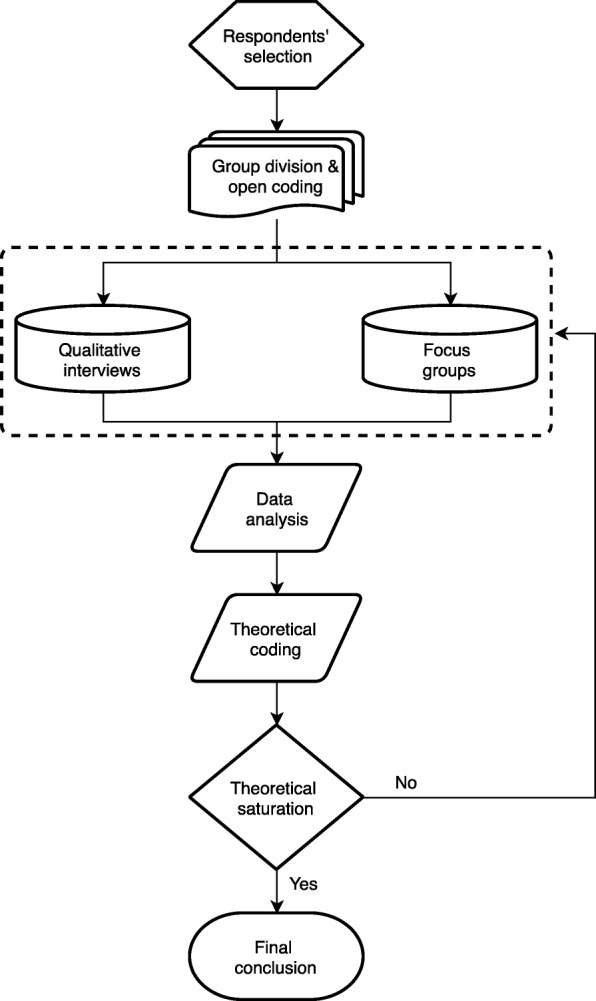
Description of the analytical approach based on GT

Taking into account the exploratory nature of the study, our analytical approach follows the constructionist perspective based on Grounded Theory (GT) described in Fig. 1 [21]. The focus groups and interviews were entirely recorded and transcribed. Consent was explicitly obtained from all participants to use audio-recorders. The materials resulting from the interviews and focus groups were included in a database created using QSR NVivo 11 qualitative analysis software by two experienced researchers. By means of this software, the information was codified following an inductive method based on constant comparison [22]. The thematic outline of the interviews and focus groups provided the initial structure that enabled the subsequent homogenisation and comparability of collated information. However, new emergent topics and analytical dimensions were selected based on the common criteria of the six researchers who participated in data gathering and analysis. In this document, the issues have been systematically organised and studied according to the vision of two fundamental groups: a) professionals in the healthcare system and the social sector; and b) the general population from different socioeconomic groups.

The consolidated criteria for reporting qualitative research (COREQ) [23] was finally used to report important aspects of the research team, study methods, context of the study, findings, analysis and interpretations.

### Ethical considerations

This study has been conducted according to the principles expressed in the Declaration of Helsinki. Ethical approval for the present study was obtained from the Centre for Andalusian Studies Committee, Seville (Spain). Written informed consent was obtained from all the participants, and the confidentiality of the information provided by the participants was ensured. All materials were stored securely to guarantee anonymity.

## Results

### Perceptions of professionals from the health system and social services

In this section, we analyse the discourse of focus groups 4 and 5, as well as the semi-structured interviews held with health professionals: a GP, an NGO manager, a caregiver, and a university professor, who is an expert in the field of public health (I1 to I4).

Participants working in health and social services or related sectors focused their discourse concerning the impact of the financial crisis on three main issues: (1) the capacity of families to cope with expenses aimed at fulfilling basic necessities such as food and health; (2) issues related to mental health; and, (3) the role of the Andalusian public healthcare system. The three topics spontaneously appeared in the participants’ discourses of both focus groups. In general terms, topics 1 and 2 are perceived as a direct consequence of the financial crisis on the health of Andalusians, whereas the last one (the impact of the crisis on the public health system) is understood to be an element that will affect the health of the region’s population indirectly and probably in the mid-long term.

#### The impact of the crisis on the capacity of families to cover necessities such as food and health

When talking about the impact of the financial crisis on the Andalusian population, professionals tended to focus on issues related to diet and difficulties accessing certain medical treatments. The participants’ discourse on this matter was quite homogeneous. On the one hand, they perceived a reduction in the quality and variety of the diet of many families, especially in the consumption of meat and fish, fruits and vegetables and fresh products in general. According to the GP:

*People buy less fruit and vegetables during times of crisis, with the repercussions this has on their health. They eat worse. It may also be a problem of family education in terms of how one should eat (I1).*

In line with this perception, professionals note that additional help is needed for those families whose members follow specific diets or treatments: gluten intolerance or a diabetic diet; children who have allergies and atopic skin; or infants fed with formula milk. According to I4, an academic expert on public health:

*A healthy and varied quality-based diet is not that cheap. For many families it is cheaper to feed their children with processed food and sadly it seems that children like it more […] and then the problem of infant obesity arises (I4).*

Participants highlighted an increased difficulty in accessing certain medications that were subsidised before the crisis, and which currently are either no longer subsidised or require co-payment. This especially affected some highly recommended medications or even ones prescribed by doctors, such as some vaccines for children. This also affected long-term medical treatments. As a result, many users end up abandoning them, due to the impossibility or permanent difficulties paying for such medical treatments. Professionals from both the health and social intervention sectors reported difficulties among low-income families in terms of accessing health products, specialised treatments or services that are intended to improve people’s health and quality of life, such as glasses, hearing aids, oral treatments and all kinds of orthopaedic devices, especially those for the elderly and people with disabilities.

*Families that had financial difficulties before the crisis already had problems getting dental treatment, glasses,* etc. *[…]*. *Now the situation is worse, it’s increased with the financial crisis (Man, 45 years old, FG4).*

Professionals considered that the most affected, in terms of health, were always those in socio-economically disadvantaged groups. They highlighted the situation of low-income individuals who were already in a low SES before the crisis, but in particular the situation of people who lost their jobs during the crisis. I4 describes the problem as follows:

*Imagine you own a couple of Mercedes [cars], a house in a good area of Seville and a chalet on the beach, and 1 day you wake up to see you have no money coming in, that your boss can’t keep paying you and unexpectedly you find yourself out on the street. And now tell your wife, tell her you can’t pay the mortgage... and see if your kids understand this... You try to find a new job and the HR guys tell you that you’re too old to work where you want to, that they’re looking for other types of profile... and you’re out on the street (I4).*

Additionally, professionals identified four groups the crisis has severely impacted in terms of health: (1) the elderly; (2) severely disabled individuals; (3) children; and (4) immigrants. Firstly, a deterioration in the health of the elderly has been observed, which is related, on the one hand, to poor consumption of medicine (e.g. self-medication) and inadequate eating habits (e.g. junk food or low consumption of fresh products) and, on the other hand, to the reduction of human resources and social/health services that were previously aimed at these collectives. Secondly, severely disabled people have seen their benefits cut, and subsequently their capacity to access to high quality home care. This situation has also affected the elderly, who in some cases have passed away while waiting for the pension or assistance that they had the right to receive. Thirdly, the effect of the crisis on children was considered to be negative in essence, in particular in the mid and long-term:

*A child is not going to die of starvation because they have stopped having breakfast, but they might not be able to pay as much attention at school, a fact that in the long term can impair achievement at school … And we know that education is a crucial social determinant of health […]*: *higher levels of education can contribute to the self-management of health without the need for high income […]*. *However, the high levels of child poverty in Andalusia, probably among the highest in the EU, will have an impact on health spending in the future. It is inevitable. Poorly educated children today will likely increase the rate of future adults having unhealthy habits and poor health (I4).*

Finally, the case of immigrants who have seen their health coverage reduced was also mentioned by professionals. On average, this social group lives with fewer resources than the native population, and after the financial crisis with fewer social and healthcare rights as well.

#### The impact of the crisis on mental health and related issues

Mental health was one of the most mentioned and analysed issues by nearly all the professionals. The general idea expressed by the participants was that the crisis has had a particular impact on the population’s mental health because unemployment has played a crucial role in the rise of illnesses associated with emotional moods (anxiety, panic attacks and depressive disruptions) as well as heart disease, principally arrhythmia and tachycardia episodes.

*The economic crisis doesn’t kill you directly, but it can reduce your future life-expectancy … continuous stress associated with long-term unemployment can unleash a depression that will eventually reduce your quality of life as well as your physical health […]*. *We have also experienced an increase in suicide rates during this crisis (I4).*

Similarly, professionals directly associated the declined in quality of life with the poor mental health of the population, considering that they have less leisure time for family, fun and holidays, which seems to have a direct impact on the psychological well-being of individuals and families. As expressed by the GP:

*In young patients we are seeing lots of anxiety disorders, depression and sleep disorders as a result of changes in their way of life and family structure. In older people we see problems related to a reversal of roles: for example, adults with families who have had to move back in with their parents. In these cases, patients who had already retired and had an acceptable quality of life have to maintain their kids and grandkids with their pensions, and pay bills, cook... All of this is reflected in mental health: anxiety, depression and restlessness (I1).*

Mental health problems were described as the precursor of somatisations and other physical health problems such as *‘migraines, digestive issues, allergic reactions, hives, psoriasis breakouts and sleep disorders’* (I1), but consequences were also found with regard to couples’ relationships and sexuality:

*Because of the loss of a job, relationship problems start to appear, issues related to cohabitation as well as sexuality. For instance, many healthy young men with no previous medical issues seek medical advice on issues related to erectile dysfunction (I1).*

#### The impact of the crisis on the role played by the Andalusian public healthcare system

The professionals interviewed shared the same view regarding the decline in the general quality of the healthcare system in Andalusia, which was found to be associated with the financial crisis and was referred to as an “involution” compared to previous years. Problems in the Andalusian healthcare service, such as the closure of health services, the reduction of average hospitalisation time, or the lack of personnel, were also expressed by participants. The reduction in the number of professionals in the public healthcare system and the increasing pressure on them to tend to more people in less time:

*Our system and our managers ask us for more and more immediacy, for us to check on more patients in less time. To solve more issues […]* *we are witnessing an era of more requests, with fewer staff and higher requirement levels (Woman, 52 year old, FG5).*

The closure of some services and hospital floors has led to the oversaturation of accident and emergency (A&E) units:

*Hospital floors are closed during certain times of the year based on staff reductions, while emergency rooms are full and don’t have enough beds […] and since we need additional space, discharges have to be faster (Man, 41 years old, FG5).*

Service closure has also increased bureaucratisation and waiting lists, both in primary care and in specialised care, such as for routine diagnosis tests and surgical interventions. Some problems encountered also related to increasing morbidity and mortality:

*Cuts can kill when the system doesn’t have the capacity to, for instance, manage or prioritise the care of patients who require urgent interventions (I4).*

In Andalusia, the public health system is predominant, so private healthcare is mainly used by people who can individually cover the costs of private medical insurance. Therefore, the decline in public healthcare would potentially affect all users, though less so in the case of social groups able to complement public healthcare benefits with private medical insurance:

*People who can pay for medical insurance can be diagnosed before others. […] In fact, the [public healthcare] doctor recommends that you go visit a private doctor (Woman, 38 years old, FG4).*

In contrast, the negative impact increased among the more vulnerable population groups and those who have suffered first-hand the direct effects of the crisis (unemployment and health problems associated with austerity policies).

With regard to the debate about public vs. private healthcare, there were different shades of opinion expressed by the participants. Most assumed that only public healthcare has suffered the impact of the crisis, whilst private healthcare continues to offer “the best and fastest service” (FG4). Nevertheless, one of the participants reports that private healthcare has indirectly suffered from the crisis. According to I1, a GP:

*I believe that private healthcare has suffered from the crisis more than the public healthcare system. Companies used to give their employees medical insurance, and a lot of the cutbacks that companies made were on medical insurance (I1).*

Finally, participants agreed that the indirect effect of the crisis on the health of the Andalusian population should be analysed prospectively. They considered that the *great effects* or the *major impact* of the crisis will be better observed in the future, i.e. in the mid and long term; that is, once variations in socioeconomic conditions have crystallised, and once the effects of downwards social mobility processes can be better perceived.

*Income, education, a person’s occupation, lifestyle (doing sport, eating healthily), risky behaviours (smoking, drinking alcohol …*), *health … All these factors go hand in hand, and when one of them fails, for some reason, the others are likely to fail as well (I4).*

### Perceived effects of the crisis on health by socioeconomic groups

In this section we analyse the impact of the financial crisis on the health of Andalusians, based on information from three different socioeconomic groups. Firstly, the situation of the socially vulnerable and socially excluded groups will be described, and then the effects of the crisis on people in more advantageous socioeconomic positions will be set out.

#### Vulnerable groups and groups suffering from social exclusion

When asked about the impact of the crisis on the health of Andalusians, most participants mentioned that the problem for people of a low SES was the difficulty they faced acquiring certain resources that are necessary for the maintenance of good health: (1) lack of a balanced diet; (2) difficulty accessing certain medications; and (3) the neglect of dentistry and ophthalmologic expenses. Firstly, the participants reported that their diet lacked protein (i.e., fish and meat) because of the increase in prices. This situation was particularly highlighted in households with children:

*It’s not just eating chickpeas, or pasta with tomato … Children have to eat yogurt, children and us, but especially children (Woman, 39 years old, FG1).*

Sometimes paediatricians recommend vaccines that are not financed or only partially financed by the public health system; so many families encountered difficulties buying these vaccines for their children. In addition, the purchase of basic medications for common illnesses had also become a problem for families that were socially vulnerable and excluded:

*When your child gets sick, you see that you don’t have a lot of medications, and you cannot afford something as simple as a syrup costing 3 euros, and you can’t afford it […]. You have to turn to someone who might help you to buy what you or your children need (Woman, 45 years old, FG1).*

However, this issue did not exclusively affect the most disadvantaged groups and population living at risk of poverty. In fact, dentistry and ophthalmologic expenses had even become unaffordable for middle-low and middle-class groups. Indeed, these expenses were particularly considered to be a luxury that cannot always be afforded:

*About 4 or 5 years ago my back tooth was broken and I have been in severe pain for several months […] and here I am with the same pain and some tablets as no dentist is financed by social security (Woman, 39 years old, FG1).*

This situation of material deprivation was perceived to have negative consequences on physical health (as in the case of toothache mentioned previously), but also especially on mental illness. Most of the socio-economically vulnerable people participating in this research suffered from anxiety and depression issues and, therefore, the vast majority received medication to treat these mental health conditions. These psychological problems were also extended to their social life, in particular to family and marital relations as well as childcare:

*About my health, yes, I am going through a tough time with my nerves; we had to separate for a while. Possibly it was my fault, because of my anxiety, my nerves. It affected me and I couldn’t control it. I had become aggressive towards him. Now I have had treatment for my anxiety and I am currently feeling a little better (Woman, 25 years old, FG1).*

Finally, financial stigma was also mentioned, which was associated with not being able to follow other people’s routines and therefore being excluded from daily activities, or with not being able to get a job and therefore being degraded at a lower personal status level.

#### Middle social class groups

For this group, the effects of the crisis on their health were focused specifically on mental health. They acknowledged that the progressive deterioration of working conditions often push them toward despair, a lack of motivation and feelings of psychological distress:

*When you feel that pressure, that you need to pay the bills, and if a bank holiday comes and I don’t work, I don’t earn any money … If I get sick I don’t work, I don’t earn any money, and with what I earn I don’t have enough to live on … So psychologically, you do feel bad. I am working all day long, and what do I have? (Woman, 50 years old, FG2).*

In contrast to the previous group, the interviewees from this group did not mention the possible effects of the crisis on their physical health, and even though they had suffered a drop in their household income, this reduction in money had not led to significant changes in their lifestyles. However, the middle-class group did report the lack of material resources in hospitals. They mentioned a lack of staff, which resulted in saturation of primary care consultations together with a delay in receiving medical appointments for specialist health services.

*They don’t give out nappies anymore, or infant formula, or needles for diabetic people […] Sometimes they don’t even do a basic medical check up (Woman, 45 years old, FG2)*.

But, generally speaking, the participants preferred the public healthcare system to the private option. They regretted that the public healthcare system in Andalusia was becoming worse than private healthcare. For instance, they mentioned the excessive waiting lists for interventions as well as the over saturation of healthcare staff. The participants repeatedly highlighted the need to pay for private insurance and, regardless of the economic effort it involves, private insurance allowed them to keep adequate control of specific illnesses. It is worth mentioning the case of families of limited economic means who have to take out private insurance to make sure that ill people in the family are properly cared for:

*Since you need to wait that long to be called for medical tests, I had to get private insurance for my daughter because she has had ear issues since she was a little girl. But for the rest of the family, we don’t have that insurance … we can’t afford it for all of us […] I’ve been paying for this private insurance for about 6 years now so that my daughter can be monitored by the ear specialist. It’s just crazy! (Woman, 52 years old, FG2).*

#### Upper social class groups

In contrast to the previous groups, this group did not consider that the crisis had directly affected their health, yet they were very aware of how it had impacted Andalusian society in general, especially with regard to younger population groups. They perceived themselves to be privileged as opposed to the vast majority of the population, who they considered to have been severely penalised by the crisis.

*In my immediate surroundings […] we have not really felt it [the crisis], but there is always a family member who does feel it because when a company is doing well everything is wonderful and when it goes wrong that’s when the trouble starts (Man, 53 years old, GD3).*

Consequently, the information they provided was not based on first hand effects suffered from the crisis. Yet, from an observer’s position, they focused on the analysis of the health system, even though, quite interestingly, many of the members of this group acknowledged that they did not use the public healthcare system but had private healthcare instead. That is, they did not refer to the undermining of the health system or of the care provided by health professionals, which they valued positively. On the contrary, they did refer to the fact that leave and absences are not being covered, and that this lack of healthcare professionals has led to the over saturation of staff and occasionally to a decline in the quality of the public healthcare system.

Occasionally, some of the interviewees from this group reported certain psychological issues (e.g., sadness, feelings of insecurity and uncertainty, etc.), which were more prominent amongst the young, whereas older groups are seen to be more resilient to the crisis:

*These years have been years in which we have had to take great care, and we have also seen that the people who have sunk most are younger people [...] they have sunk much more than older ones, much more. Older people always have more resources because we have lived through greater austerity; we spent more time living in poverty in our childhood (Woman, 70 years old, GD3).*

### Qualitative synthesis of main analytical dimensions and topics

To complement the analysis and provide an easy interpretation of the results, the main topics raised among the different groups studied (i.e. professionals and the general population) have been summarised according to three analytical dimensions: (1) health service deterioration (2); increasing unmet needs; and (3) poor mental health (Table 3).

**Table 3:** Main analytical dimensions and topics extracted from the analysis

		Health service deterioration	Increasing unmet needs	Poor mental health
Professionals	Healthcare professionals	- Public health *service closure* - Public health service *oversaturation* - Poor *working conditions* in public health	*- Diet and healthy habits in low-income groups* - Need for private health insurance	- Mental health issues in young population - Mental health issues in unemployed - *Sexual dysfunction* in men
	Social services professionals	- Public health *service closure* - Public health service *oversaturation*	- *Diet and healthy habits in low-income groups* - Difficulties accessing *specialised treatments and health products*	- *Mental health issues in low-income* groups - Poor *family and social relationships*
General population	High income	- Public health service *oversaturation* - Use of private health insurance	- Problem of family member, friends or neighbours	- Mental health issues in young population
	Mid income	- Public health *service closure* - Public health service *oversaturation* - Increasing *waiting lists* in health services	- Difficulties accessing *specialised treatments and health products* - Need for private health insurance	- *Mental health issues in young population* - *Mental health issues in unemployed* - *Mental health issues in women*
	Low income	- Public health *service closure* - Public health service *oversaturation* - Increasing *waiting lists* in health services	*- Diet and healthy habits in low-income groups* *- Diet and healthy habits in vulnerable groups (*e.g. *children)* - Difficulties accessing *specialised treatments and health products*	- *Mental health issues in young population* *- Mental health issues in low-income* groups - Poor *family and social relationships*

**Fig. 2:**
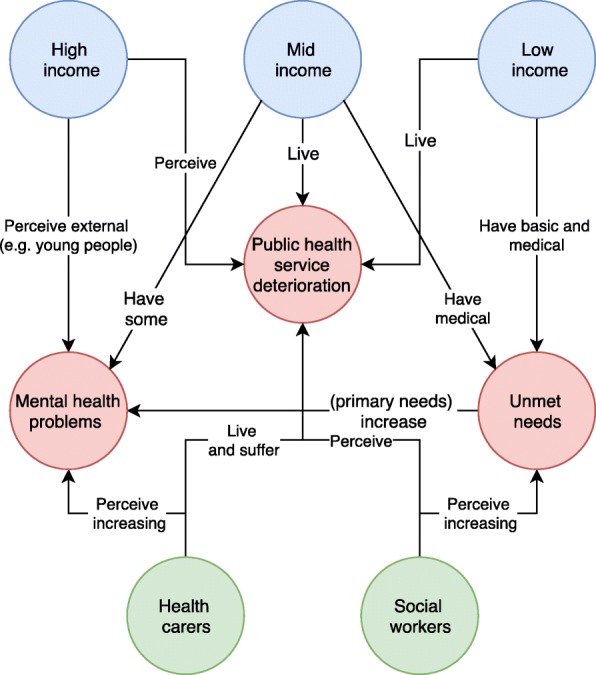
Conceptual map of analytical categories

Figure 2 represents the conceptual map that describes the relationships between the different main topics and groups of participants. Blue nodes represent the socioeconomic groups, green nodes include professional groups, and red nodes represent the main analytical categories: (1) mental health problems, (2) health services deterioration, and (3) unmet needs. Relationships between nodes are described by arrows that signal the perceptions, experiences, and/or problems of each informant group. The resulting schema was agreed by the participants in this study.

## Discussion

The financial crisis has appeared to significantly affect the public healthcare system, although its effect on each social group has not been equal. A continuum in the analysis of these discourses could be established, with one end representing the most financially vulnerable population whose discourses are characterised by first-hand experiences and a microscopic analysis of day-to-day questions, which focus on basic material needs – such as food and medication. Advancing along this continuum, the discourses of the middle and upper classes come to be less based on first-hand experiences in as much as questions that surpass material aspects – such as mental health and general wellbeing – are included, and so their analysis takes on a more macro dimension, focused on the healthcare system. Lastly, the discourse of the professionals reinforced the arguments of these groups and incorporated a reflection on the health and social policies that shape their working conditions and the life of the different social groups. For this reason, since this research considers a wide range of social actors, it provides a general description of the way in which the Andalusian population perceives the effects of the crisis on health, perceptions that in many cases are widely shared and agreed upon by the different collectives.

In line with previous studies, our work reveals some of the most notable consequences on the socially vulnerable groups and people at risk of social exclusion; that is, elderly people, minors, females, unemployed people, people with mental health issues and immigrants [7, 9, 12, 16, 17]. Additionally, the structural mechanisms that give rise to economic and health issues are outlined, which can mostly be defined by the lack of jobs and/or finance, which then has an influence on lifestyles and the material conditions of families, known as the intermediary determinants of health [24]. Although the health inequalities associated with gender have not been explicitly pointed out, it is worth emphasising that the best descriptions of families’ situations have been suggested by women who take care of dependents and children. An implicit piece of information was the dual responsibility that Andalusian women usually bear by working both inside and outside the domestic sphere. In summary, the caregiver role of females in this region needs to be considered in order to understand the higher prevalence of depressive disorders among this social group, found in previous studies [19].

The processes of downward social mobility have been found to have consequences in the short term, as families face difficulties regaining their previous living standards from before the crisis. They also have consequences in the long term for minors who have suffered the impact of the economic crisis [25, 26]. In the interviews and focus groups conducted, several family issues are mentioned, including difficulties feeding children, buying school material and medicines (e.g., vaccines). If the logic of social determinants of health is to be considered, then these barriers would have a great impact in the long run. That is, the crisis has not just affected the health of families but in particular their socioeconomic conditions, and it is known that variations in SES may contribute to the rise of health inequalities throughout a person’s life cycle [25–29]. As a result, socioeconomic problems that may affect the educational processes of children or the working conditions of the young population today might explain future health inequalities in later life [26]. It is known that low educational attainment increases the risk of poorer health, for instance through the effect of intermediary factors such as risky or non-healthy lifestyles [19]. Education favours the inclusion of the young in the job market while at the same time it teaches about them the most appropriate health behaviours (healthy diets, benefits of physical activity, and danger associated with alcohol or tobacco consumption) [30]. Consequently, as stated in previous research, education is crucial to reduce health inequalities [31].

This research shows that the crisis has had a major impact not just on families but on the whole of society and in particular on welfare state resources, which have been cut during this period. Thus, the deteriorated socioeconomic conditions left behind by the crisis (e.g., higher unemployment rates, risk of poverty and deprivation, etc.) might increase health inequalities in the future [17]. Therefore, the major enduring health inequalities found in Andalusian society, such as those associated with gender or specific age groups (e.g. the elderly or younger workers), might even be further exacerbated in the future if measures that favour health and social equity in the population are not taken [17]. In Andalusia, as in the rest of Spain, the health inequalities that prevail in Europe as a whole are reproduced [32, 33]. Inequalities due to ethnicity, gender, age, unemployment, disability and health are associated with the socioeconomic difficulties experienced by many families, as well as with their lifestyles and sociodemographic features [28]. Even though these inequalities are common to those existing in other countries, regions such as Andalusia—characterised by a weak productive sector and a highly segmented job market—are more likely to be subject to the influence of future macro-economic fluctuations as well as future inequities in health [2, 30].

For the reasons stated above, the need for comprehensive policies that go beyond health intervention is clear. Improving the health of social groups at risk of social exclusion requires not only health and social intervention but also educational measures aimed at self-empowerment and socio-occupational inclusion that would allow them to escape from their financial dependency [30]. A greater development of policies around social protection that guarantee the education of children and the minimum income for low-income families, in parallel with greater investment in preventive policies in the health sector. At the same time, it is crucial to regain the quality of public health services [5] as well as to remove the co-payment system that has a negative effect on the health of these groups [34]. Therefore, educational policies should also be developed for the social inclusion of those with fewer opportunities. Finally, there is a clear need to continue working on the development of analytical models based on the evidence—quantitative and qualitative—that would allow us to understand the complex interrelations between social determinants of health, as well as the multiple consequences of the combination of certain socioeconomic and health circumstances on specific population groups.

This paper presents certain limitations. On the one hand, despite the participation of professionals from both the health and social sectors, we could not recruit professionals from the private healthcare sector, who might have provided an additional perspective on the effect of the crisis. Secondly, the attribution of the socioeconomic position of general population groups was inferred from the residential area where individuals were recruited (low, mid and high-income neighbourhoods), and this strategy makes it impossible to recruit social class profiles from either extreme due to accessibility problems (i.e., poorest areas and wealthier areas).

## Conclusion

This qualitative study disentangles the mechanisms of social inequalities in health through the perceptions, experiences, and problems of experts and the general population that experienced the 2008 economic downturn in Andalusia. Our study provides deep insight into the health and social consequences of the recent financial crisis in the region of Andalusia (Spain) through the first-hand information of professionals from the health and social sector, and also of the general population, characterised by different socioeconomic positions. This study shows how the economic downturn has both directly and indirectly impacted the population from this region. In Andalusia, the crisis has not only affected the health and social wellbeing of low-income groups with higher unmet needs (i.e. access to specialised healthcare services, treatments, medicines, etc.), but has also led to a decline in mental health among other socioeconomic strata (e.g. mid/high SES individuals) and specific socio-demographic groups (e.g. young population).

Unlike most of the research conducted in relation to the crisis, this work reflects how the economic crisis has impacted the most vulnerable sectors in all areas of daily life. In particular, our findings highlight the need to implement integral policies that protect the health of the most vulnerable groups: elderly population, minors, females, unemployed people, people with mental health issues and immigrants. Finally, according to our findings, future policy-action plans should address mental health problems, unmet (basic and medical) needs among the socially excluded and populations at risk of social exclusion, and the progressive deterioration of the national health system.

## Data Availability

This study is based on data from the project ‘Socioeconomic Determinants of Health in Andalusia: Studying the Impact of the Economic Crisis on Andalusian Health’ (IMPACT-A) (Grant No. PRY120/14). Data may be accessed having obtained previous permission from the participants and consent from the researchers.
